# Machine learning-based glycolysis-associated molecular classification reveals differences in prognosis, TME, and immunotherapy for colorectal cancer patients

**DOI:** 10.3389/fimmu.2023.1181985

**Published:** 2023-05-05

**Authors:** Zhenling Wang, Yu Shao, Hongqiang Zhang, Yunfei Lu, Yang Chen, Hengyang Shen, Changzhi Huang, Jingyu Wu, Zan Fu

**Affiliations:** ^1^ Department of General Surgery, The First Affiliated Hospital of Nanjing Medical University, Nanjing, Jiangsu, China; ^2^ The First School of Clinical Medicine, Nanjing Medical University, Nanjing, Jiangsu, China

**Keywords:** glycolysis, colorectal cancer, molecular subtypes, tumor immune infiltration, machine learning, single-cell analysis

## Abstract

**Background:**

Aerobic glycolysis is a process that metabolizes glucose under aerobic conditions, finally producing pyruvate, lactic acid, and ATP for tumor cells. Nevertheless, the overall significance of glycolysis-related genes in colorectal cancer and how they affect the immune microenvironment have not been investigated.

**Methods:**

By combining the transcriptome and single-cell analysis, we summarize the various expression patterns of glycolysis-related genes in colorectal cancer. Three glycolysis-associated clusters (GAC) were identified with distinct clinical, genomic, and tumor microenvironment (TME). By mapping GAC to single-cell RNA sequencing analysis (scRNA-seq), we next discovered that the immune infiltration profile of GACs was similar to that of bulk RNA sequencing analysis (bulk RNA-seq). In order to determine the kind of GAC for each sample, we developed the GAC predictor using markers of single cells and GACs that were most pertinent to clinical prognostic indications. Additionally, potential drugs for each GAC were discovered using different algorithms.

**Results:**

GAC1 was comparable to the immune-desert type, with a low mutation probability and a relatively general prognosis; GAC2 was more likely to be immune-inflamed/excluded, with more immunosuppressive cells and stromal components, which also carried the risk of the poorest prognosis; Similar to the immune-activated type, GAC3 had a high mutation rate, more active immune cells, and excellent therapeutic potential.

**Conclusion:**

In conclusion, we combined transcriptome and single-cell data to identify new molecular subtypes using glycolysis-related genes in colorectal cancer based on machine-learning methods, which provided therapeutic direction for colorectal patients.

## Introduction

Recently, reprogramming of the tumor’s energy metabolism has emerged as one of the tumor’s hallmarks (1). Since the “Warburg effect” was put forth, researchers have steadily investigated the connection between tumors and glycolysis (2, 3). As is a crucial form of the transformation of energy, glycolysis converts glucose into pyruvate, eventually creating lactic acid and giving tumor cells oxygen-independent energy (4). Tumor cells can still restrict energy metabolism to glycolysis, often known as aerobic glycolysis, even in an aerobic environment (3). By reducing cell inhibition and apoptosis, glycolysis creates the conditions for tumor cell growth (5, 6). Numerous intermediate products of glycolysis serve as starting materials for the synthesis of nucleosides and amino acids, both of which aid in the production of new cells (7). Additionally, certain tumor cells fall into one of two subgroups: aerobic or hypoxic. The utilization of lactate and glucose in these two subgroups’ energy metabolisms differs from one another (8). In conclusion, glycolysis is crucial for the energy metabolism of tumors.

Studying colorectal cancer (CRC) is important due to its high incidence and fatality rates (9). Glycolysis is implicated in the development of colorectal cancer, according to increasing amounts of evidence (10). Many glycolysis-related genes, including lactate, GLUT1, pyruvate kinase M2, glyceraldehyde-3-phosphate dehydrogenase, enolase-1, lactate dehydrogenase 5, and hexokinase 2, are currently discovered to be up-regulated in colorectal cancer (11–17). Additionally, there is growing appreciation for the function of the tumor microenvironment (TME). Studies show that different areas of a tumor mass exhibit metabolic heterogeneity (18). CRC cells contain both OXPHOS and glycolysis phenotypes (19). Near the blood capillaries, the majority of CRC cells have greater OXPHOS, whereas tumor cells farthest from the blood vessels exhibit a glycolysis phenotype (20). This unexpected increase in OXPHOS is described as the “reverse Warburg effect” (10). Meanwhile, the interaction between tumor cells and other cells in the TME has an impact on metabolic remodeling. Lactate is transported from CAF to CRC cells when mono carbohydrate transporters (MCTs) are upregulated (21). A study showed the primary enzyme of glycolysis, pyruvate kinase M (PKM1 and PKM2), which is artificially highly expressed in stromal cells, has the potential to encourage tumor cell proliferation and invasion (22). While naive T cells depend on OXPHOS for energy supply, activated T cells require glycolysis (23). All of the evidence provided shows that glycolysis is essential for controlling the interaction between tumor cells and TME.

Immunotherapy has gained significant traction in recent years for the treatment of advanced solid tumors, including colorectal cancer. Especially, Immune checkpoint inhibitors (ICIs) are beneficial for treating patients with metastatic colorectal cancer who have mismatch-repair-deficient (dMMR) or microsatellite instability-high (MSI-H). Unfortunately, ICIs have not yet been effective in CRC patients with mismatch-repair-proficient (pMMR) or microsatellite-stable (MSS) or low microsatellite instability (MSI-L) (24, 25). Additionally, acquired resistance has emerged as one of the impediments to immunotherapy. Hence identifying new CRC subtypes and screening novel treatment targets are crucial. Targeted therapy for glycolysis has gained popularity due to the role glycolysis plays in CRC (4). For instance, compound 2-deoxyglucose (2-DG), a glycolysis inhibitor, can lessen cell invasion (26). LND, another glycolysis inhibitor, can also make chemotherapy treatments more effective and stop the growth of several tumor cells, including CRC (10, 27). However, due to the different functions of glycolysis in various cell types and the heterogeneity of carbohydrate metabolism in tumor cells, targeted medications for glycolysis are not that effective. Therefore, it is crucial to investigate the subtypes of glycolysis in CRC to uncover potential therapeutics. Currently, despite some studies reporting CRC’s glycolytic signature employing several genes and long non-coding RNAs (28, 29), there is no research focusing on the gene-based molecular subtypes of glycolysis in CRC. In this study, we used the unsupervised clustering method to categorize each CRC patient using genes associated with glycolysis. The characteristics of each glycolysis-associated cluster’s (GAC) clinical, genomics, TME, and enrichment pathway were then addressed. We also examined the distribution and function of each GAC-like subtype in each cell after mapping each GAC type to the single-cell data. Based on the findings above, we integrated the results of single cell and bulk RNA-seq to develop a model that predicts the GAC of each CRC patient and verified it, whose role in treatment was also explored.

## Materials and methods

### Patient population and bulk RNA expression acquisition

Five colorectal cancer data were involved in this study, including TCGA-COAD/READ, GSE39582, GSE38832, GSE17538, and GSE14333. Corresponding clinical features and RNA-seq data were achieved from UCSC Xena (https://xenabrowser.net/) and the Gene Expression Omnibus (GEO) (https://www.ncbi.nlm.nih.gov/geo/). We excluded patients with OS and DFS less than 30 to ensure reliability in accordance with previous studies (30–32). Particularly, we employed TCGA data as the training set and GEO data as the verification set. For this study, we obtained the characteristic of 1603 CRC patients, the baseline of which was shown in **Table S1**. Besides, we downloaded expression profiles and clinical data from the two cohorts (IMVigor210, GSE78220) for seeking the role GAC predictor played in immunotherapy. The batch effect has been removed by the “Combat” algorithm.

### Consensus clustering analysis

198 Glycolysis-related genes were acquired from the MSigDB Team (HALLMARK_GLCOLYSIS) (**Table S2**). To further examine the various expression patterns of glycolysis-related genes in CRC, we performed consensus clustering using the k-means method with the”ConsensusClusterPlus” package to classify individuals with CRC (33). We set 80% sampling each time and 1000 iterations to ensure the consistency of the clustering process. The optimal number of the clustering was determined by the consensus heatmap and cumulative distribution function (CDF) curves. Kaplan–Meier curves were drawn to exhibit the prognosis of each glycolysis-associated cluster (GAC) using “survival” and “survminer” packages (34). The same procedure was conducted once more by using DEGs between the GACs, which was to acquire gene clusters.

### Gene set variation analysis and consensus molecular subtype classification

Supported by the “GSVA” package, we utilized the GSVA method to explore the differences in pathways enriched by each cluster. Gene sets (C2.cp.kegg, C2.cp.reactome, C2.go.bp, and hallmark gene sets) were downloaded from the MSigDB Team v2022.1. “limma” package was used to screen significant pathways (adjusted p-value< 0.05). The symbol pathways of each cluster were condensed and shown as a heatmap. We performed GSVA in bulk and single-cell level. Besides, we applied the CMScaller package to identify each CRC patient’s CMS attribution.

### Single-sample gene set enrichment analysis

With quantification of pathway enrichment in each patient, ssGSEA enables the comparability of various pathways in each sample. The “GSVA” package was utilized in the procedure. To better reveal the clinical and CMS heterogeneity, we plotted a heatmap to visualize the differences. Related pathways were achieved from the “CMScaller” package. Based on Lee et al.’s study (35), markers of 31 cell types with log2FC >0.25 were delivered to the ssGSEA scoring system. Besides, marker genes in Charoentong’s study were utilized (36).

### Single-cell data preparation

We obtained GSE132465 for single-cell analysis. Corresponding RNA-seq and annotation data were downloaded in the GEO database. Clinical information of 23 CRC patients was acquired from the original article (35). Based on the “Seurat” package, we carried out the preliminary processing of the scRNA-seq data following the criterion of Lee et al.’s study (genes detected in each cell, mitochondrial gene expression). 23 tumor samples were extracted and we further run the “Harmony” function to integrate the scRNA-seq. The top 30 dimensions were selected while processing the t-SNE.

### Identification of GAC at single cell level

We further mapped the consensus clusters (GAC) to the scRNA-seq to investigate their function at cellular level. With the “FindAllMarkers” function, we identified the marker genes of each GAC in bulk-seq data (LogFC >0.5) and then submitted them to the “AddModuleScore” function in scRNA-seq data. Each cell was given a score, and the cluster with the highest score was used to determine the cell’s final annotation result. The annotation was shown in **Table S3**. Combing the original and GAC annotation of each cell, we obtained a new glycolysis-related cell subtype such as “Epithelial-G1”. Then in scRNA data, we collected the marker genes of each cell subtype (**Table S4**). Additionally, glycolysis-related genes signature was also demonstrated by the “AddModuleScore” function.

### Prognosis analysis based on glycolysis-related cell subtype

With the marker genes of glycolysis-related cell subtype obtained using the “FindAllMarkers” function, we selected those with the top 50 ranks in log_2_FC (If less than 50, follow the existing options) and applied them to GSVA analyses in bulk-seq data. Based on the GSVA score, the prognosis-related cell subtypes were found using Cox regression analysis, and the most significant cell subtypes were identified by combining the findings from various cohorts using the “rma” function of the “metafor” package.

### Pseudotime trajectory analysis and cell-cell communication analysis of glycolysis-related epithelial cell subtypes

By creating the evolutionary trajectory between cells, pseudotime analysis predicts the transformation of cells over time. First, we extracted epithelial cells and performed the same quality control, integration, and dimension reduction process. Then we conducted pseudotime analysis on the processed data with the “monocle3” package. Additionally, cell communication analysis was completed with the “CellChat” package.

### Assessment of TME among GACs using different algorithms

To depict the landscape of tumor immune infiltration with bulk-seq data, we performed three algorithms: ssGSEA, Cibersort, and Estimate. We calculated the ssGSEA score of 31 cell types in Lee et al.’s research (35). Charoentong’s cell types were also described by ssGSEA algorithms. We further calculated the proportion of each immune cell with Cibersort analysis. Estimate algorithm contains three scores: Stromal score, immune score, and combined score. All the above analyses were conducted in TCGA and GEO datasets for the robustness of the results.

### Genome-related analysis and cancer stem cell index

In this part, we focused on the mutation of the tumor, copy number variation (CNV), methylation, microsatellite stability, and cancer stem cell index. The somatic mutation data and CNV files were downloaded from the GDC TCGA database. Supported by the “maftools” package, we drew waterfall plots to demonstrate the somatic mutation of COAD and READ patients in TCGA. TMB was quantified into log(TMB), termed as TMB score. Downloaded by “TCGAbiolinks”, CNV and methylation were exhibited by lollipop and violin plots, respectively. MSI status was plotted using a graph of proportion. Subsequently, Utilizing the data from Progenitor Cell Biology Consortium (PCBC), we trained the model of mRNAsi prediction and further applied it to calculate the CSC index of our samples. The relationship between GAC and CSC index was discussed.

### Construction and validation of GAC predictor using four machine learning methods

The TCGA cohort served as our training set, and the GEO cohorts served as our validation set. To increase the reliability of the results at the macro (Bulk-seq) and micro (scRNA-seq) levels, the genes used to predict GAC were determined to be the intersection of DEGs of GAC and marker genes for glycolysis-related single-cell subtypes that have prognostic implications. Four machine learning methods were conducted to select important genes, including least absolute shrinkage and selection operator (LASSO) regression, random forest (Boruta), extreme gradient boosting (XGBoost), and support vector machine (SVM) (37–40). The R package we used contained “glmnet”, “randomForest”, “Boruta”, “XGBoost”, “e1071”, and “caret”. We used these important genes to identify the GAC of each patient. The final model, termed as “GAC predictor”, was decided as multinomial logistic regression and constructed by the “multinom” function of the “nnet” package. We estimated which GAC each patient might belong to using the “prediction” function, and we chose the GAC with the highest probability. “pROC” and “caret” packages were used to evaluate the results by calculating AUCs, accuracy, sensitivity, and specificity. We further tested the “GAC predictor” in a similar way on the validation sets. The model was shown as follows:

$${\text{Model}}_{\text{GAC}3}=\;\sum_{1}^{i}\left(\text{EstiGAC}3\text{i }*\text{ ExpGenei}\right)$$

$${\text{Model}}_{\text{GAC}2}=\;\sum_{1}^{i}\left(\text{EstiGAC}2\text{i }*\text{ ExpGenei}\right)$$

$${\text{Model}}_{\text{GAC}1}=\;0\;(\text{reference group})$$

$${\text{P}}_{\text{GAC}3}=\text{exp}\left({\text{Model}}_{\text{GAC}3}\right)/\left[\text{exp}\left({\text{Model}}_{\text{GAC}1}\right)+\text{exp}\left({\text{Model}}_{\text{GAC}2}\right)+\text{exp}\left({\text{Model}}_{\text{GAC}3}\right)\right]$$

$${\text{P}}_{\text{GAC}2}=\text{exp}\left({\text{Model}}_{\text{GAC}2}\right)/\left[\text{exp}\left({\text{Model}}_{\text{GAC}1}\right)+\text{exp}\left({\text{Model}}_{\text{GAC}2}\right)+\text{exp}\left({\text{Model}}_{\text{GAC}3}\right)\right]$$

$${\text{P}}_{\text{GAC}1}=\text{exp}\left({\text{Model}}_{\text{GAC}1}\right)/\left[\text{exp}\left({\text{Model}}_{\text{GAC}1}\right)+\text{exp}\left({\text{Model}}_{\text{GAC}2}\right)+\text{exp}\left({\text{Model}}_{\text{GAC}3}\right)\right]$$

EstiGAC3 and EstiGAC2 represented the estimate of multinomial logistic regression. ExpGene represented the expression of the gene. P represented the probability that each patient belongs to each GAC.

### Treatment-related analysis

To excavate the treatment of each GAC, we obtained potential therapeutic compounds related to each GAC based on typical marker genes using Cmap analysis (https://clue.io/). For each GAC, the 10 compounds with the lowest enrichment score, which were considered antagonistic drugs, will be presented in the form of a heatmap. Besides, to gauge each GAC’s drug sensitivity, we computed the semi-inhibitory concentration (IC50) values of common medicines using the “pRRophetic” package.

### Statistical analysis

Wilcoxon rank-sum test was applied to show the difference between the two groups. Wilcoxon signed-rank test was used in two paired groups. Kruskal-Wallis H test was performed to compare three or more groups. Dunn test was used for multiple comparisons. Chi-square test was used in the proportion test. The Pearson test was used to identify correlations of the data. The log-rank test method was employed to analyze survival data. All of the statistical analyses were conducted using R 4.1.3 (p< 0.05).

## Results

### Classification of glycolysis-associated molecular clusters in colorectal cancer

**Figure 1:**
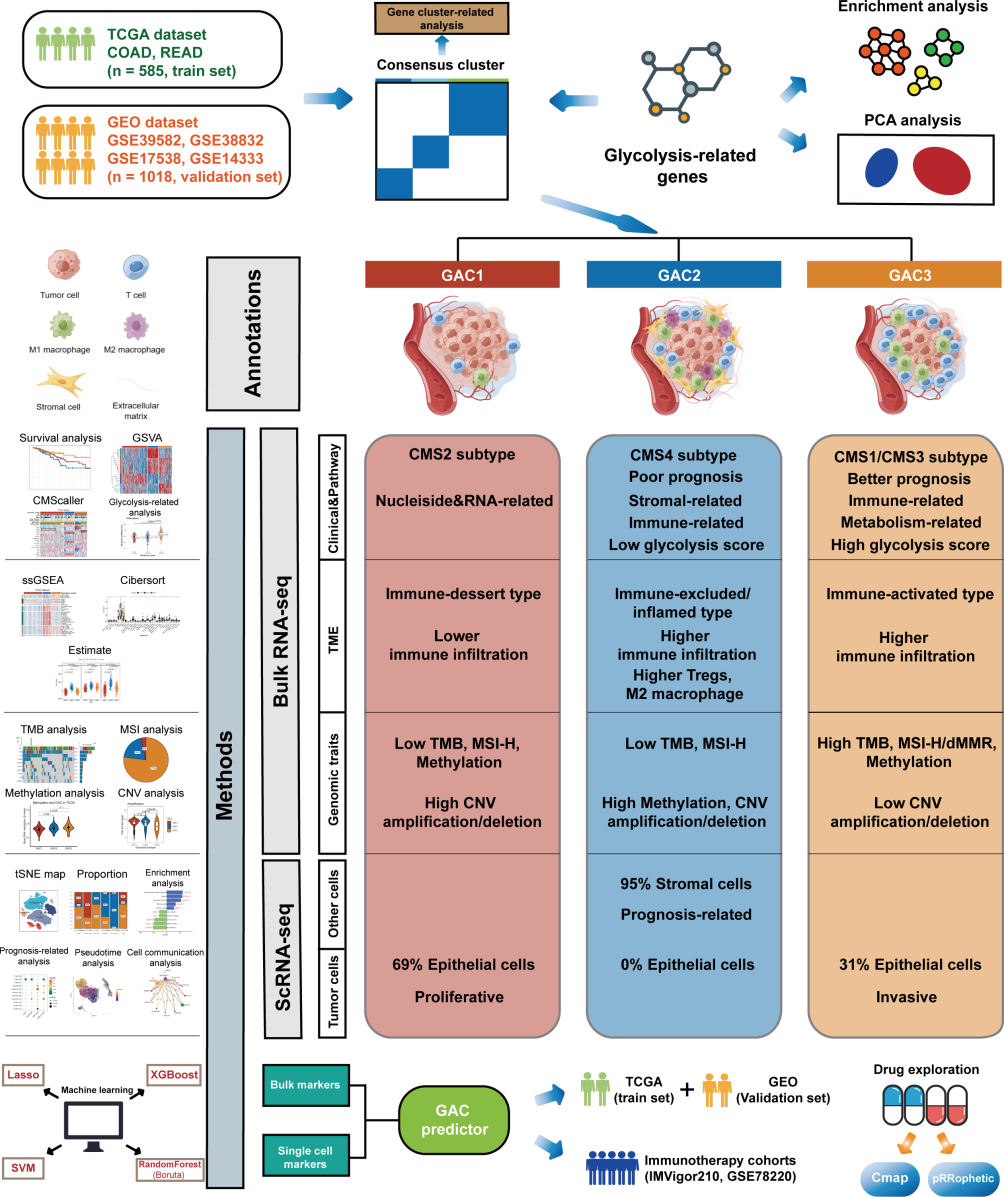
The whole flow chart and the summarized features of each GAC.

**Figure 2:**
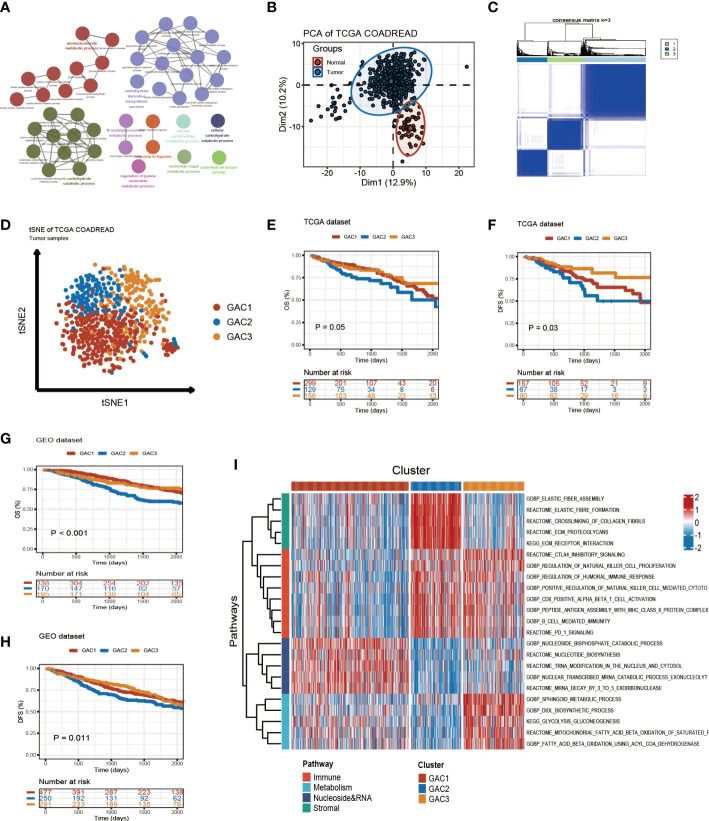
Glycolysis-related molecular subtypes in colorectal cancer. **(A)** Glycolysis-related pathways presented by GO enrichment analyses using metascape. **(B)** Principal component analysis utilizing glycolysis-related genes to separate tumor from normal tissue. **(C)** Consensus clustering identifying three clusters with different expression pattern of glycolysis-related genes. **(D)** tSNE plot visualizing the three GACs with obvious differentiation. **(E-H)** Survival curves of OS and DFS for the GACs in TCGA and GEO datasets. **(I)** GSVA analyses characterizing the biological process of the three GACs. Red to blue represents the range of enrichment from high to low.

The whole follow chart was shown in **Figure 1**. We collected 198 glycolysis-related genes from the MSigDB Team (HALLMARK_GLCOLYSIS) for further study. Employing GO enrichment analysis, we found these genes were characterized by the metabolic process of carbohydrates and derivatives (**Figure 2A**). The comparison of glycolysis-related genes between normal and tumor tissues from TCGA COAD/READ was visualized with a PCA map. The two main components indicated that glycolysis-related genes were effective at separating tumors from normal tissue (**Figure 2B**). To investigate the latent role these genes played in oncogenesis, we used consensus clustering to categorize CRC patients (TCGA and GEO datasets). Three glycolysis-associated clusters were identified, namely GAC, according to **Figures 2C** and **S1**. To explore the distribution of each GAC, t-SNE analysis was employed and showed significant GAC heterogeneity (**Figure 2D**).

### The clinical discrepancy among GACs

**Figure 3:**
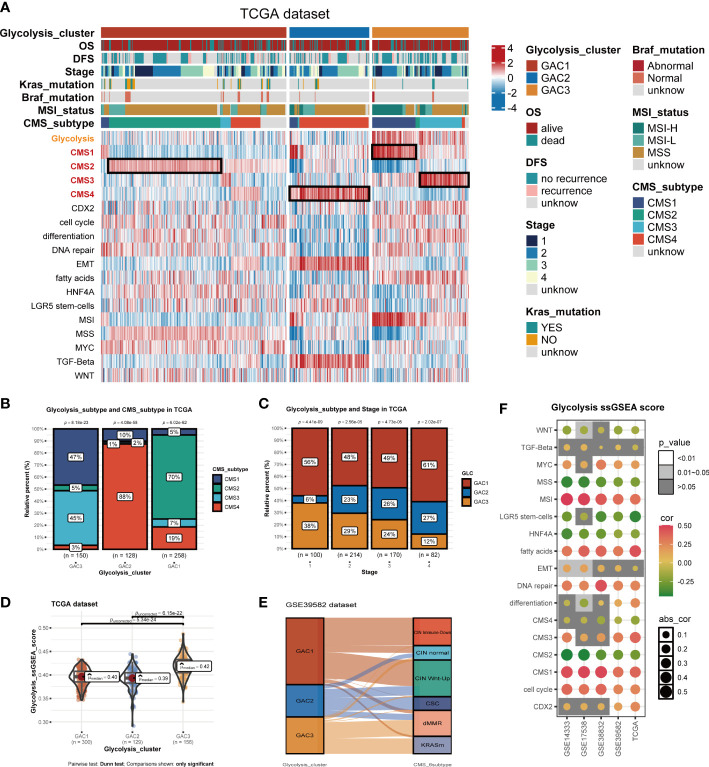
Biological activities and clinical features of the three GACs. **(A)** The heatmap comprehensively assessed each GAC’s biological and clinical parameters in TCGA dataset. Notable CMS subtypes with high correlations to GACs were represented by the black mark box. **(B)** Bar charts showing CMSs’ proportion of each GAC with chi-square test. **(C)** Bar charts showing each stage’s proportion of each GAC with chi-square test. **(D)** The distribution of glycolysis score acquired from ssGSEA in the three GACs. Comparations were conducted by Dunn test. **(E)** Alluvial plot exhibiting the molecular subtype attribution of GACs in GSE39582. **(F)** The association between glycolysis and various pathways visualized by a bubble chart.

Built on GAC clustering, we discussed the corresponding clinical features of each GAC. First, we plotted survival curves of overall survival (OS) and disease-free survival (DFS) for three GACs and found GAC2 was in an unsatisfactory prognosis (TCGA-OS: p = 0.05; TCGA-DFS: p = 0.03; GEO-OS: p< 0.001; GEO-DFS: p = 0.011, log-rank test) (**Figures 2E–H**). Moreover, the relationship between clinical stage and GAC indicated that the proportion of GAC2 increased with advancing stage and GAC3 on the opposite, which was consistent with the poor prognosis of GAC2 (**Figures 3C**, **S2D**). According to **Figures 3A**, **S2A**, and **Table S1**, GAC1 has a larger proportion of Kras mutations, while GAC3 has a higher proportion of Braf mutations and MSI-H status. Besides, GAC2 possessed the youngest age distribution. The clinical characteristics of GACs were described in this section as being heterogeneous: 1. GAC2 had a poor prognosis with advanced stage and younger trend; 2. GAC3 held a favorable prognosis with an early stage and higher mutation probability. 3. GAC1 was in the middle position of various clinical features.

### Biological differences and features of each GAC

Utilizing “GSVA” algorithm, we elaboratively chose four types of phenotype (immune-related, metabolism-related, nucleoside&RNA-related, and stromal-related) as the exhibition in the form of a heatmap (**Figure 2I**). The pathways contained “C2.cp.kegg”, “C2.cp.reactome”, and “C2.go.bp”. With the “limma” package, differential pathways were identified. GAC1 was enriched in pathways connected to nucleosides. GAC2 was associated with stromal and immunological functions. Immune and metabolic traits were present in GAC3. Built on this, the relationship between GAC and CMS was worth discussing. Afterward, we created a heatmap to display the relationship between GAC and CMS (**Figure 3A**). Surprisingly, we found GAC1 and GAC2 overlap mostly with CMS2 and CMS4, respectively. While in patients with GAC3, CMS1 and CMS3 occupied an equal and largest proportion (**Figures 3A, B** **)**. We further validated these findings using the classical pathways of the “CMScaller” package. “EMT” and “TGF-Beta” pathways, associated with CMS4, were enriched in GAC2, which was a convincing illustration (**Figures 3A**, **S2A, B**). We quantified the glycolysis pathway with ssGSEA and discovered a high expression in GAC3 (**Figures 3A, D**, **S2A, E**). Additionally, we found that glycolysis was negatively connected with WNT, MSS, LGR5 stem cells, HNF4A, CMS2, and CMS4, and positively correlated with MSI, fat acid, DNA repair, CMS1, CMS3, and cell cycle (**Figure 3F**). A displayed alluvial diagram of GSE39582 further demonstrated that GAC1 flows to CIN immune-down and CIN Wnt-up, GAC3 flows to dMMR and KRASm, and GAC2 flows to the remainder (**Figure 3E**). Conclusively, GAC1 shared the most features of CMS2. GAC2 was the most similar to CMS4, and GAC3 share the dual features of CMS1 and CMS3. Same discoveries were found in the GEO dataset (**Figure S2**).

### Mapping glycolysis-associated clusters onto CRC cells at scRNA-seq level

**Figure 4:**
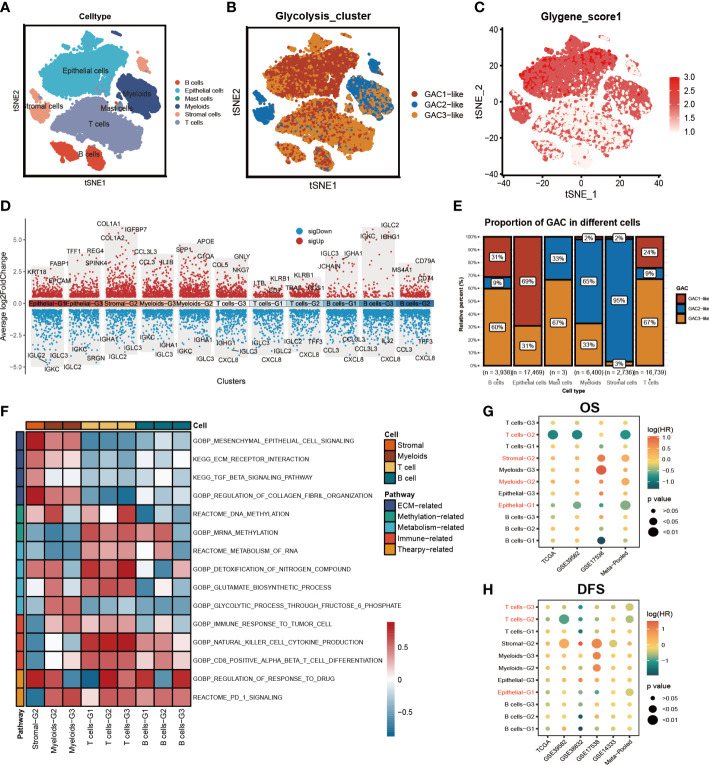
The characteristics of the tumor microenvironment in each GAC at single cell level. **(A)** The distribution of each cell type visualized by tSNE map. **(B)** Mapping GACs into each cell and the distribution of GACs at scRNA level. **(C)** The dispersion of glycolysis score in the tSNE map. **(D)** Marker genes of each cell subtype. **(E)** The bar graph displaying the percentage of each GAC in various cells. **(F)** GSVA analysis using a heatmap to depict each cell subtype’s typical biological processes. **(G, H)** Meta-analysis determining the cell subtypes related to prognosis (OS, RFS).

On the basis of bulk-seq, we attempted to construct this GAC classification on individual cells. Through preprocessing of GSE132465, 23 tumor samples were integrated to perform further analysis. Cell type annotation was based on Lee et al.’s study and visualized by a t-SNE map (**Figure 4A**). With “AddModuleScore” utilized, we successfully mapped three GAC-like subclusters onto single cells (**Figure 4B**). Additionally, the glycolysis-related gene signature (glygene) was confirmed in **Figure 4C**, demonstrating its main presence in myeloid, stromal, and epithelial cells. The proportion of each GAC in each cell was then examined after we added the GAC-like subclusters to each cell type (**Figure 4E**). Interestingly, we discovered that epithelial cells were filled only with GAC1-like (69%) and GAC3-like (31%). Though T and B cells shared GAC characteristics with epithelial cells, GAC3-like was presented in higher concentrations than GAC1-like in these cells, which makes them distinct from epithelial cells. Stromal cells were almost GAC2-like (95%), consistent with **Figures 1I**, **2B**. Myeloids contain 65% GAC2-like and 33% GAC3-like. The marker genes of each cell subtype were depicted by a combined volcano plot (**Figure 4D**, **Table S4**). GSVA analysis revealed distinct biological pathways among GAC-like subclusters (**Figure 4F**). Apparently, pathways of drug response, DNA methylation, and glutamate metabolism were enriched in myeloids-G2, while myeloid-G3 was on the opposite. T cell-G2 has a favorable response to drugs. B cell-G1, 2 had higher mRNA methylation. Importantly, we filtered the most clinically relevant GAC-like subclusters uniting prognosis (OS and DFS), the marker genes of which were responsible for later GAC predictor development. With meta-pool analysis showing the significance of T cells-G2, T cells-G3, Stromal-G2, Myeloids-G2, and Epithelial-G1, we identified these five GAC-like subclusters as independent prognostic factors (**Figures 4G, H**).

### Analysis of the two GAC-like subclusters in epithelial cells

**Figure 5:**
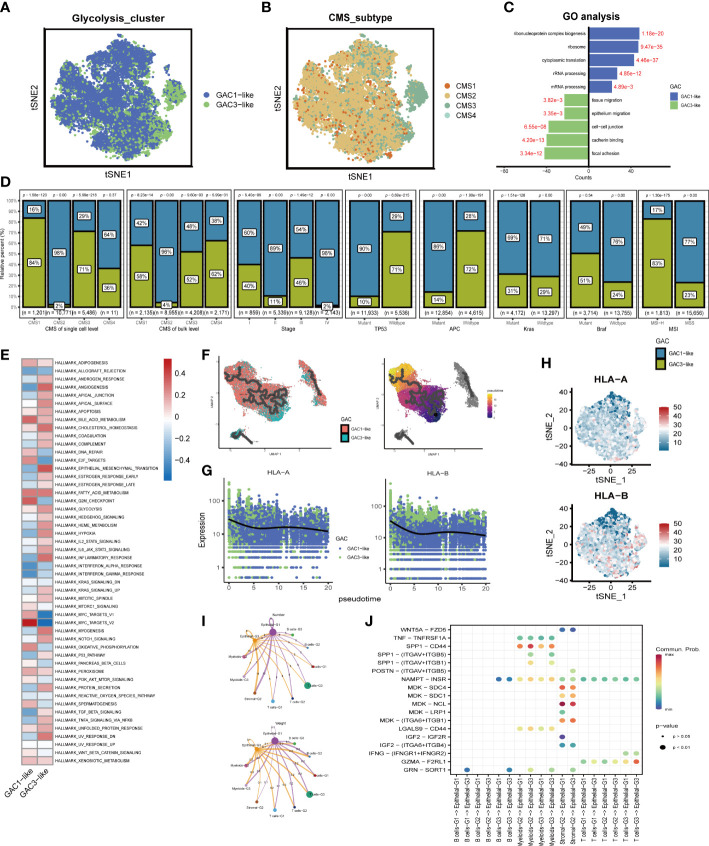
The features of the two types of GAC-like tumor cells at single cell level. **(A)** The distribution of the GAC1-like and GAC3-like tumor cells in tSNE map. **(B)** The distribution of each CMS subtype in tSNE map. **(C)** GO analysis showing the feature of each GAC-like tumor cell. **(D)** The bar charts summarizing the clinical features of each tumor cell subtypes. (CMS subtype at scRNA-seq and bulk RNA-seq level, stage, the mutation of TP53, APC, Kras, and Braf, and MSI status) **(E)** GSVA analysis revealing the hallmarks of each GAC-like tumor cell. **(F)** Pseudo-time analysis showing the development of the two GAC-like tumor cells. **(G, H)** The expression pattern of HLA-A and HLA-B in the two cell types. **(I)** Cell-cell communications described by numbers and weight of intercellular connections. **(J)** Cell-cell communications described by receptor-ligand analyses using a bubble chart.

We subsequently chose epithelial cells to analyze their characteristics. The distribution of GAC-like subclusters and CMS subtypes was presented in the t-SNE map (**Figures 5A, B**). Discovery was found that the corresponding relationship between CMS and GAC-like subclusters was comparable to the bulk-seq’s, demonstrating the relationship’s robustness. Through GO enrichment analysis we found that epithelial-G3 was enriched in the feature of metastasis, whereas epithelial-G1 was mostly associated with RNA biological process (**Figure 5C**). Likewise, by GSVA analysis we displayed 50 hallmark signatures in the two types of epithelial cells, demonstrating GAC3-like’s anaerobic traits and GAC1-like’s ability to up-regulate oxidative phosphorylation (**Figure 5E**). The results of both enrichment analyses showed that GAC1-like epithelial cells proliferated and that GAC3-like epithelial cells invaded. In addition, the study on the clinical characteristics of the two epithelial subtypes showed varieties. For the CMS subtypes, CMS2 was GAC1-like at both the scRNA and bulk-RNA levels, while a significant fraction of GAC3-like was found in CMS1 and CM3, which was in line with previous results. For stage, patients in the middle and late phases of CRC demonstrated an increased percentage of GAC1-like epithelial cells. For mutation, GAC1-like epithelial cells harbored high levels of TP53 and APC mutations, the rest site not significant. For MSS, most GAC3-like cells have MSI-H features (**Figure 5D**). According to pseudo-time analysis, epithelial cells will transition from being GAC3-like to being GAC1-like (**Figure 5F**). Meanwhile, HLA-A and HLA-B were primarily found in GAC3-like cells, suggesting that these cells had a stronger propensity for immunological responses (**Figures 5G, H**). Finally, cell communication analysis was used to investigate the impact of non-tumor cells on the tumor epithelium. The frequency of linkages between Sromal-G2 and Myeloid-G2 and epithelial cells was substantially higher, but Epthelial-G3 was more significantly impacted by B cells-G2 (**Figure 5I**). A bubble plot specifically showed the ligand-receptor network, from which we could tell SPP1-CD44 was noteworthy in myeloids-epithelial interactions. CD44 was considered to promote stemness and invasiveness in colorectal cancer (41). MDK ligands were associated with multiple receptors to regulate the interaction between stromal and epithelial cells. GZMA-F2RL1 was identified in the communication of T cells-G3 and Epthelial-G3 (**Figure 5J**). Conclusively, the proliferative type, epithelial-G1, and the invasive type, epithelial-G3, were both regulated by TME.

### Correlation between GAC and tumor microenvironment

**Figure 6:**
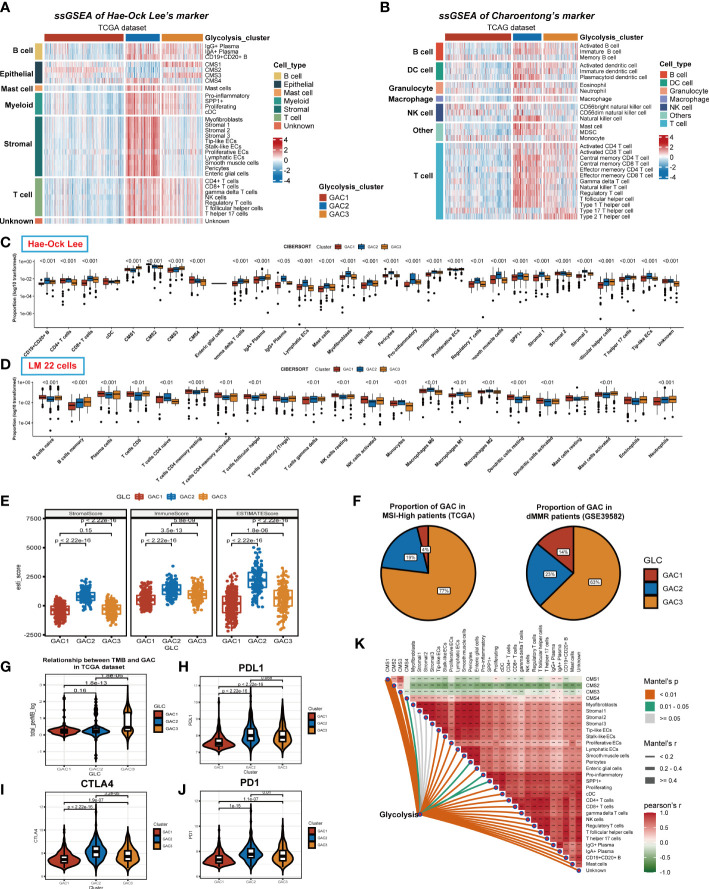
Immune infiltration analyses of the three GACs at bulk RNA-seq level in TCGA dataset. **(A, B)** ssGSEA analyses based on markers of Hae-ock Lee and Charoentong revealing the expression of TME cells of the GACs. **(C, D)** Cibersort analysis depicting the percentage of each cell in GACs based on markers of Hae-ock Lee and LM 22 cells. The proportion was converted to log10 format for better visual effects. Statistical analyses still used the unconverted original data source. **(E)** Estimate algorithm calculating stromal, immune and overall score of the GACs. **(F)** The pie chart showing the proportion of MSI-H and dMMR in each GAC. **(G)** Violin chart exhibiting the TMB score of each GAC. **(H-J)** The expression of PD1, PDL1, and CTLA4 in each GAC. **(K)** The relationship between TME cells and glycolysis. (*p<0.05, **p<0.01, ***p<0.001).

We previously discussed the heterogeneity of GAC in TME at single-cell level, and in this part, we would go back to the bulk level. First, we collected two groups of TME cell signatures (Lee’s and Charoentong’s) for ssGSEA analysis. Same as the scRNA level, we found stromal cells mainly converged in GAC2 in bulk data. B cells, Myeloid cells, and T cells were aggregated in GAC2 and 3. The expression of epithelial cells were in line with the results in scRNA. Remarkably, GAC2 possessed more regulatory T cells (Tregs) (**Figures 6A**, **S3A**). In Charoentong’s signature, macrophage, T, and B cell expression trends were similar to Lee’s observed findings (**Figures 6B**, **S3B**, **S4A, B**). The Cibersort algorithm calculated the expression proportion of each cell. In Lee’s cell signatures, the fraction of proliferative ECs was the highest in TME, proliferating macrophage the second, among which GAC2 occupies the lowest and highest, respectively (**Figures 6C**, **S3C**). In LM cell signatures, the same phenomenon was seen in macrophages, while T cells were the second proportion of all cells and the lowest in GAC2. It was noteworthy that the proportion of M2 macrophage in GAC2 was greater (**Figures 6D**, **S3D**). According to the Estimate methodology, GAC2 and GAC3 had higher stromal and immune scores than GAC1 (**Figures 6E**, **S3E**). To predict the responses of different GACs to immunotherapy, we collected MSS, dMMR, TMB, and PD-1 information. The outcome was significant: GAC3 had more percentage of MSI-High and dMMR (**Figure 6F**), and achieved a higher TMB score (**Figure 6G**). While GAC2 had relatively higher expression of PD-1, PDL-1, and CTLA-4 (**Figurse 6H–J**, **S3F, H**). The correlations of Lee’s cells were presented in **Figures 6K** and **S3I**, in which we showed the relationship between glycolysis and each cell. We concluded the same results in the GEO dataset (**Figure S3**). GAC1 had the least immune cell infiltration, GAC2 possessed a higher amount of stromal and immune cells but also a large proportion of tumor-promoting cells (Tregs, M2 macrophage), and GAC3 had a higher immune cell infiltration. Summarily, GAC1 was comparable to the immune-desert type, GAC2 to an immune-inflamed/excluded type, and GAC3 to an immune-activated type.

### Identification and description of gene clusters

**Figure 7:**
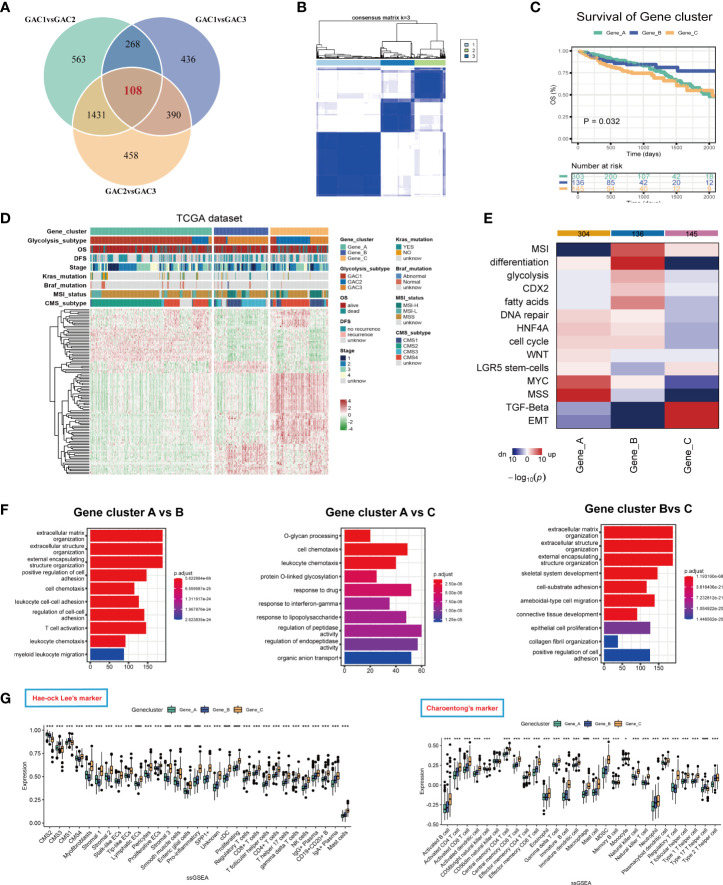
Description of glycolysis-related gene clusters. **(A)** Intersection of the DEGs of the three GACs. **(B)** Consensus cluster performed using intersected DEGs. **(C)** Survival curves showing the prognosis of each gene cluster (log-rank test). **(D)** A heatmap characterizing the clinical features and expression pattern of the gene clusters. **(E)** Enrichment analysis of each gene cluster based on CMScaller. **(F)** GO analysis revealing the biological process of the gene clusters. **(G, H)** Immune infiltration-based ssGSEA analysis on the gene clusters using markers of Hae-ock Lee and Charoentong.

We identified gene clusters to investigate the intrinsic causes for the existence of DEGs between GACs (**Table S6**). To obtain the final DEGs, the intersection of the DEGs between each pair of GACs was employed (**Figure 7A**, **Table S5**). Then Three clusters, referred to as gene clusters, were obtained after 108 genes were submitted to consensus clustering (**Figure 7B**). In terms of clinical characteristics, we discovered that gene cluster B had a significant prevalence of GAC2 and a bad prognosis (**Figure 7C**). These 108 genes’ clustering expression patterns and additional clinical traits were displayed in **Figure 7D**, showing discrepancies among gene clusters. In terms of enrichment analysis, gene cluster A has upregulated MSS and MYC. In gene cluster B, differentiation, MSI, glycolysis, and fatty acids were highly enriched. TGF-Beta and EMT were activated in gene cluster C (**Figure 7E**). According to GO analysis, gene clusters B and C were linked to extracellular matrix synthesis and epithelial cell proliferation, whereas gene cluster A was linked to T cell activation and O-glycan process (**Figure 7F**). We discovered that the gene cluster C group has strong expression in the majority of TME cells using two ssGSEA signatures. Immune cells from gene cluster B were relatively activated (**Figures 7G, H**). All considered, the clinical, biochemical, and immunological features of GACs were supported by gene clustering.

### Exploration of TMB, CNV, methylation, and CSC index between GACs

**Figure 8:**
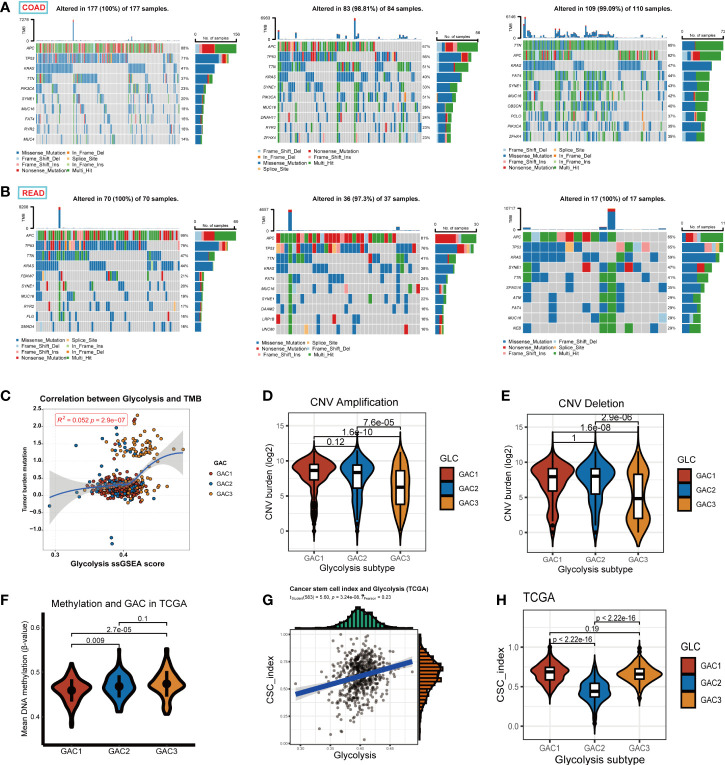
Exploration of the properties of each GAC at genomic level. **(A, B)** Waterfall Plots revealing the genes with top 10 mutation frequency in each GAC of COAD and READ. **(C)** The relationship among glycolysis score, tumor mutation burden score, and GACs. **(D, E)** Copy number variation (amplification, deletion, log2 transformed) of each GAC. **(F)** The relationship between methylation and GACs in TCGA dataset. Methylation was calculated in the form of the mean value of B-value. **(G)** The relationship between glycolysis score and CSC index (r =0.23, p<0.001). **(H)** Violin chart representing the association between GACs and CSC-index.

This section focused on the investigation of tumor stemness and genomics. We displayed the first 10 mutant genes of each GAC in COAD and READ using waterfall plots. In COAD, the mutation of GAC1 and GAC2 were similar, while in GAC3 we did not find a high mutation rate of TP53. In READ, we detected a high mutation rate of FATA4, especially in GAC2. APC, TP53, TTN, and KRAS were the genes with the top mutation frequency (**Figures 8A, B**). Besides, we noticed that the TMB score and glycolysis score were favorably connected and GAC3 has high levels of TMB and glycolysis attributes (**Figure 8C**). Meanwhile, GAC3 possessed a lower copy number variation, which indicated an opposite of pro-tumor effect (**Figures 8D, E**) (42). The overall methylation of GAC1 was lower than the other two (**Figure 8F**). Through CNV calculation, we found CSC index was in a positive correlation with the glycolysis score, with GAC2 occupying the lowest CSC index (**Figures 8G, H**). Taken together, we recognized GAC1 as a low methylation type, GAC2 as a low CSC type, and GAC3 as a low CNV and high TMB type.

### Machine learning-based construction of GAC predictor and description of GAC Predictor-related genes (GP genes)

**Figure 9:**
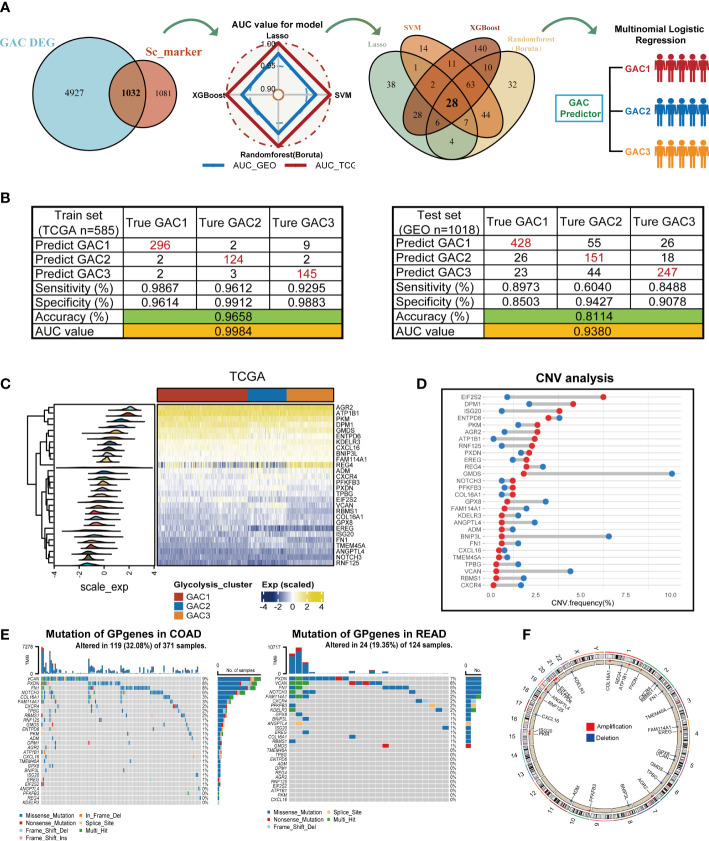
Construction of the GAC predictor using four machine learning methods and characteristics of the selected gene variable. **(A)** Flow chart describing the process of establishing the GAC predictor. **(B)** Verification of the GAC predictor in sensitivity, specificity, accuracy, and AUC value in both the train and validation set. **(C)** Heatmap showing the expression of the 28 GP genes in trainset. **(D)** CNV frequency of the GP genes. **(E)** Somatic mutation landscape of the GP genes in COAD and READ patients. **(F)** Circle plot showing the location of the GP genes in chromosome.

In this part, we constructed a prediction model for the GAC status of each patient using four machine-learning techniques. We referred to TCGA as the training set and GEO as the validation set. The whole process was shown in **Figure 9A**. To ensure the reliability of the results, we used clinically significant genes at both bulk and single-cell levels for subsequent analyses. Thus, the intersection of GAC DEGs and marker genes of significant cell subtypes (**Figures 4G, H**) was obtained. To retain the genes that have the most influence on the prediction results, we used Lasso, SVM, Randomforest (Boruta), and XGBoost for screening (**Figure S5**; **Table S7**). The radar graphic demonstrated that the four methods’ AUC values in the test set and training set were high. We again intersected the genes filtered by the four machine-learning methods to acquire a final gene set, named GP genes. Subsequently, GP genes were used for multinomial logistic regression to construct the final GAC predictor (**Table S8**). With the GAC predictor applied for verification, in both training and validation sets we gained high levels of AUC and accuracy values (training set: AUC = 0.9984, accuracy = 0.9658; validation set: AUC = 0.9380, accuracy = 0.8114) (**Figure 9B**). We further conducted several analyses for GP genes. The heatmap showed the expression of GP genes in order (**Figure 9C**). Genomics-related research on GP genes, including TMB and CNV analyses, was performed. We discovered EIF2S2, DPM1, and ISG20 as CNV amplification, GMDS, BNIP3L, and VCAN as CNV deletion (**Figure 9D**). All GP genes were presented in the chromosome loop diagram (**Figure 9F**). By TMB analysis, we identified VCAN, PXDN, FN1, and NOTCH3 as genes with high mutation frequency in both COAD and READ (**Figure 9E**).

### Treatment strategies for each GAC and immunotherapy cohorts for GAC predictor validation

**Figure 10:**
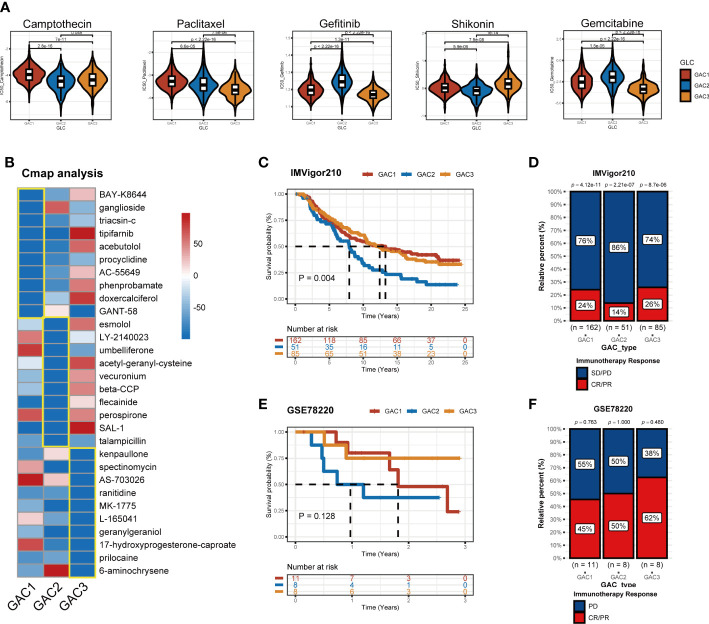
Application of GACs in treatment area. **(A)** IC50 of several common chemotherapeutic drugs in each GAC. **(B)** Cmap analysis revealing potential small compounds for the treatment of each GAC. **(C, D)** Validation of IMVigor210 immunotherapy cohort in prognosis of different GACs. **(E, F)** Validation of GSE78220 immunotherapy cohort in prognosis of different GACs.

Chemotherapy and targeted medications increase the survival rate of colorectal cancer with advanced stages. However, some patients are still achieving little benefits or developing treatment resistance (43). Hence it is necessary to formulate a personalized drug treatment plan for each CRC patient. Based on the significant clinical and biological differences among the three GAC, we assumed that each GAC had its own appropriate treatment and then investigated the sensitivity and antagonistic effects of typical chemotherapeutic medicines as well as possible small molecule medications in three GACs. The “pRRophetic” package and Cmap analysis were used to implement the treatment prediction for each GAC. Drug sensitivity, in the form of IC50, was produced as **Figure 10A** showed. For GAC1, camptothecin and paclitaxel were more sensitive. Gefitinib and gemcitabine had higher sensitivity to GAC2. Shikonin had more therapeutic potential for GAC3. Additionally, we conducted Cmap analysis to acquire potential compounds resisting each GAC. Stronger compounds’ efficacy resulted from lower scores. 30 compounds were exhibited in **Figure 10B** to provide treatment strategies. To test the role of GAC clustering in the immunotherapy cohorts, we obtained two datasets for verification (IMVigor210 and GSE78220). With the GAC predictor applied In IMVigor210, we found GAC2 had a poor prognosis (p = 0.004, log-rank test), along with immunotherapy response (**Figure 10C, D**). In GSE78220, the same conclusion was achieved that GAC2 was with unfavorable OS rate (p = 0.128, log-rank test). Even though GSE78220’s p-value is not significant, each GAC’s survival pattern is clearly visible (**Figure 10E, F**). Overall, we explored GAC’s potential compounds and validated the GAC predictor in the field of immunotherapy.

## Discussion

As research into cancer has progressed in depth, it has also been discovered that cancer possesses aberrant metabolic characteristics. The evidence of the Warburg effect demonstrates that aerobic glycolysis has a significant part to play in tumor growth. An illustration of this is hexokinase 2 (HK2), which is prevalently expressed in tumors and is able to gain access to ATP in the inner mitochondrial membrane through voltage-dependent anion channels, thus enhancing glucose metabolism and preventing cell death (44). Glycolysis with its high rate of output not only produces energy rapidly but also furnishes tumor cells with the necessary components for the assembly of numerous biological macromolecules (7). Lipids, nucleotides, and amino acids, which are converted from glycolysis, provide the raw materials for cell growth and division (45, 46). Furthermore, glycolysis is associated with numerous carcinogenic signaling pathways and genetic mutations. Abnormal activity of the Wnt signaling may interfere with the activity of Pyruvate Dehydrogenase Kinase 1 (PDK1), thus preventing the connection between glycolysis and the TCA cycle, consequently hampering OXPHOS (47). HK2, a key enzyme of glycolysis, can be induced by p53. In the absence of glucose, mutant p53 can reduce AMPK signaling, resulting in an increase in aerobic glycolysis (48). Additionally, the focus is gradually being directed to the metabolic remodeling of TME. Pyruvate is converted by glycolysis to lactate, which makes the tumor microenvironment acidic. The immunological effectiveness of NK cells is reduced by this acidic environment (49). During activation, CD8T cells, NK cells, and M1 cells all display significant glycolytic characteristics, whereas Treg cells and M2 cells favor the usage of OXPHOS (50, 51). According to these findings, tumors may have aberrant energy consumption habits that help them thrive ecologically and impair immunotherapy (52). As an important promoter of tumor progression, glucose metabolism has been extensively exploited to overcome the bottleneck of immunotherapy (53). Glycolysis-targeted drugs in combination with immunotherapies have shown to be highly effective in CRC treatment, according to growing preclinical evidence (54–56). All these demonstrate how important glycolysis is for the study and treatment of colorectal cancer.

In this study, we utilized unsupervised clustering to identify three clusters of CRC patients, termed as GACs, based on the expression of genes related to glycolysis. The three GACs had entirely different clinics, biological processes, immune infiltration, and genomic traits when viewed from bulk-seq perspective. Bioinformatics methods were used to assign a distinct GAC composition to each cell group from a single-cell view. It was discovered that each cell type with a distinct GAC type possessed its unique traits. Finally, we built the GAC predictor by integrating bulk and single-cell RNA-seq data to identify the GAC attribution of each CRC patient.

Initially, when analyzing bulk RNA-seq data, we discovered that genes related to glycolysis were capable of distinguishing between tumor and normal samples. We then divided these genes into three glycolysis-associated clusters *via* unsupervised clustering algorithm in CRC patients. Different characteristics of various GACs were revealed by multiple enrichment analyses (GSVA, ssGSEA). From the perspective of biological pathways, GAC1 tended to be active in cell cycle and replication, and its relatively elevated WNT and MYC pathways were consistent with the CMS2 subtype, as evidenced by its high proportion of the CMS2 subtype. GAC2 had the greatest CMS4 ratio, a tendency to be more stromal-type, and was heavily enriched in EMT and TGF-Beta pathways. With greater MSI and fatty acid metabolic pathways and high proportion levels of CMS1 and 3 at the same time, GAC3 has a tendency to be immunologically and metabolically enriched (**Figures 2I**, **3A**). Each GAC’s enrichment pathway exhibits a high degree of concordance with the enrichment pathway of the GAC-associated CMS subtype described in the previous article (57). From a clinical perspective, the prognosis of GAC2 was worse while that of GAC3 was better, both of which were consistent with the corresponding CMS subtypes. From the perspective of the TME, we used algorithms like ssGSEA, cibersort, and ESTIMATE to investigate the variations in the immune microenvironment of GACs. Similar to the immune desert type, GAC1 is obviously devoid of immune cell infiltration. In contrast, GAC2 and GAC3 exhibit significant immune infiltration properties. GAC2 demonstrated a condition of stromal infiltration, and more tumor-promoting immune cells (Treg, M2 macrophage) were present, indicating that it might be the immune-inflamed/excluded type. A Study revealed that M2 macrophages can promote the invasion and metastasis of CRC cells by releasing exosomes that carry mi-RNA (58), This supported the opinion in the study that GAC2, which was infiltrated by M2 macrophages, enriched in metastasis-related pathways, such as EMT and TGF-Beta. GAC3, however, was more like the immune-activated type. Comparable to Emilie Picard’s article (59), this result mapped the immune microenvironment of each GAC. From a genomics perspective, APC mutations were more prevalent in GAC1, whereas TTN mutations were more prevalent in GAC3 (**Figures 8A, B**). GAC1 resembled a classical type more due to the accumulation of the APC/KRAS/TP53 mutation (60). High TTN mutation rate was related to high TMB status (61), which GAC3 both possessed (**Figure 8C**). In terms of prospects for immunotherapy, GAC3 demonstrated a more favorable reaction than GAC1 and 2. Clinical trials have demonstrated that immunotherapy is effective for MSI-H/dMMR CRC patients (62, 63). Particularly, in GAC3 there existed simultaneous MSI-H/dMMR status and better prognosis, the correlation between which has been verified in many clinical studies and meta-analyses (64, 65), that is, MSI status has a better prognosis than MSS status in stage II/III colorectal cancer, attributed to the lower probability of recurrence of MSI status (66). When compared to GAC1, GAC2 and 3 expressed more PD-1, PDL-1, and CTLA-4, while GAC3 had more MSI-H, and dMMR phenotypes concurrently (**Figures 6F, G**), indicating that GAC3 had immunotherapeutic potential. Meanwhile, our results supported the presence of elevated immune checkpoint molecule expression in CMS1 and 4 (57, 67, 68). As expected, GAC3 showed a favorable prognosis in the immunotherapy cohort of bladder and melanoma cancer (**Figure 10**). From the perspective of therapeutic drugs, shikonin had a high sensitivity to GAC3 and a study has demonstrated that it inhibits the proliferation of colorectal tumor cells when used as a treatment (69). Meanwhile, gemcitabine was more effective against GAC2, and the fact that it can cause apoptosis in oxaliplatin-resistant cells further supports its potential to act as an inhibitor of colorectal cancer (70).

When analyzing scRNA-seq data, we uncovered that glycolysis predominantly occurred in tumor cells, stromal cells, and myeloid cells (**Figures 4A, C**), which aligned with the findings of prior research (71, 72). Interestingly, our investigations indicated that stromal cells mainly belong to the GAC2 subtype, while tumor cells were predominantly GAC1 and GAC3 (**Figures 4B, E**), affirming our previous bulk RNA-seq study’s determination that GAC2 was of stromal-type origin. The stroma, where cancer-associated fibroblasts interact with cancer cells to promote invasion, metastasis, EMT, and drug resistance, is crucial in the development of cancer (73). Since there are only two GAC-like subtypes of tumor cells, we investigated this more thoroughly and used enrichment analysis (GO and GSVA) to discover that GAC1-like tumor cells had a higher propensity for division and replication while GAC3-like tumor cells were more likely to invade and adhere. Pseudo-time analysis showed a possible transition of tumors from GAC3-like to GAC1-like, which was worth exploring. Besides, cell communication analysis found significant interactions between myeloid and stromal cells on tumor cells, with stronger signaling from receptor-ligands of SPP1-CD44, MDK-NCL, and MDK-SDC4. Glioma, pancreatic cancer, and intrahepatic cholangiocarcinoma have been reported to possess the SPP1-CD44 axis that is immunosuppressive and pro-tumor (74–76). Although it was discovered that midkine (MDK) was increased in colorectal cancer (77) and that nucleolin (NCL) was linked to DNA and RNA metabolism and proliferation (78), the precise relationship between the two has not yet undergone experimental verification and merits more investigation. Syndecan-4 (SDC4) is connected to a poor prognosis and the invasion of colorectal cancer cells. All these scRNA-related results enhanced the conclusion derived from bulk RNA-seq data and excavated the character of tumor cells of different GAC-like subtypes.

Meanwhile, our study has some limitations. First, the data were based on retrospective studies obtained from public databases, and there was a lack of validation of a prospective cohort originating from our research center. Second, only bioinformatics algorithms were used to anticipate rather than experimentally validate the biological properties of tumor cells with different GAC-like subtypes. Additionally, the sample size used in this study was insufficient, therefore the GAC predictors’ sensitivity and accuracy needed to be enhanced by adding samples and validation.

## Conclusions

In summary, we identified three glycolysis-related molecular subtypes of CRC, whose characteristics of the clinic, genomics, and immune infiltration were discussed based on the integration of bulk and single-cell RNA-seq data. We simultaneously created predictive models to forecast the GAC subtype in each sample and offered medication suggestions for treatment.

## Data availability statement

The datasets presented in this study can be found in online repositories. The names of the repository/repositories and accession number(s) can be found in the article/**Supplementary Material**.

## Author contributions

ZW, YS and HZ contributed to the study equally. ZF, ZW and YS conceived and designed the manuscript. ZW, HZ and YL wrote the manuscript. ZW, CH, JW, YL, YC, HZ and YS analyzed the data and drew the figures. ZF and HS helped with the manuscript and data review. All authors contributed to the article and approved the submitted version.

## Supplementary material

Supplementary Figure 1 Consensus clustering of the glycolysis-related genes. **(A)** The heatmap of consensus clustering with k from 2 to 8. **(B)** The CDF threshold curve. **(C)** The delta area.

Supplementary Figure 2 The validation of clinical and biological pathway characteristics in GEO datasets. **(A)** The heatmap comprehensively assessed each GAC’s biological and clinical parameters in GEO datasets. **(B)** The boxplots visualizing each GAC’s pathway enrichment scores (* p<0.05, ** p<0.01, *** p<0.001). **(C)** The relationship between GACs and CMS type in GEO datasets. **(D)** The relationship between GACs and stage in GEO datasets. **(E)** The distribution of glycolysis score in each GAC.

Supplementary Figure 3 The validation of TME features of each GAC in GEO datasets. **(A, B)** ssGSEA analyses based on markers of Hae-ock Lee and Charoentong revealing the expression of TME cells of the GACs in GEO datasets. **(C, D)** Cibersort analysis depicting the percentage of each cell in GACs based on markers of Hae-ock Lee and LM 22 cells in GEO datasets (* p<0.05, ** p<0.01, *** p<0.001). **(E)** Estimate algorithm calculating stromal, immune and overall score of the GACs in GEO datasets. **(F–H)** The expression of PD1, PDL1, and CTLA4 in each GAC in GEO datasets. **(I)** The relationship between TME cells and glycolysis score.

Supplementary Figure 4 Further exploration of immune infiltration of GACs in both TCGA and GEO datasets. **(A, B)** The boxplots quantifying the ssGSEA score of immune cells in each GAC for the validation. **(C, D)** The proportion of GACs in each immune-subtype.

Supplementary Figure 5 The visualization of four machine learning methods contributing to the selection of important genes. **(A, B)** The importance of the selected features using random forest (Boruta) methods. **(C)** The linear cost selection of SVM methods. **(D, E)** The Lasso method filtering variables. **(F)** The importance of the selected variables using XGBoost method.
