# Supplementary figures and images for: Machine learning-based glycolysis-associated molecular classification reveals differences in prognosis, TME, and immunotherapy for colorectal cancer patients
Source: Front Immunol. 2023 May 5;14:1181985. doi: 10.3389/fimmu.2023.1181985 (PMC10203873; doi:10.3389/fimmu.2023.1181985)

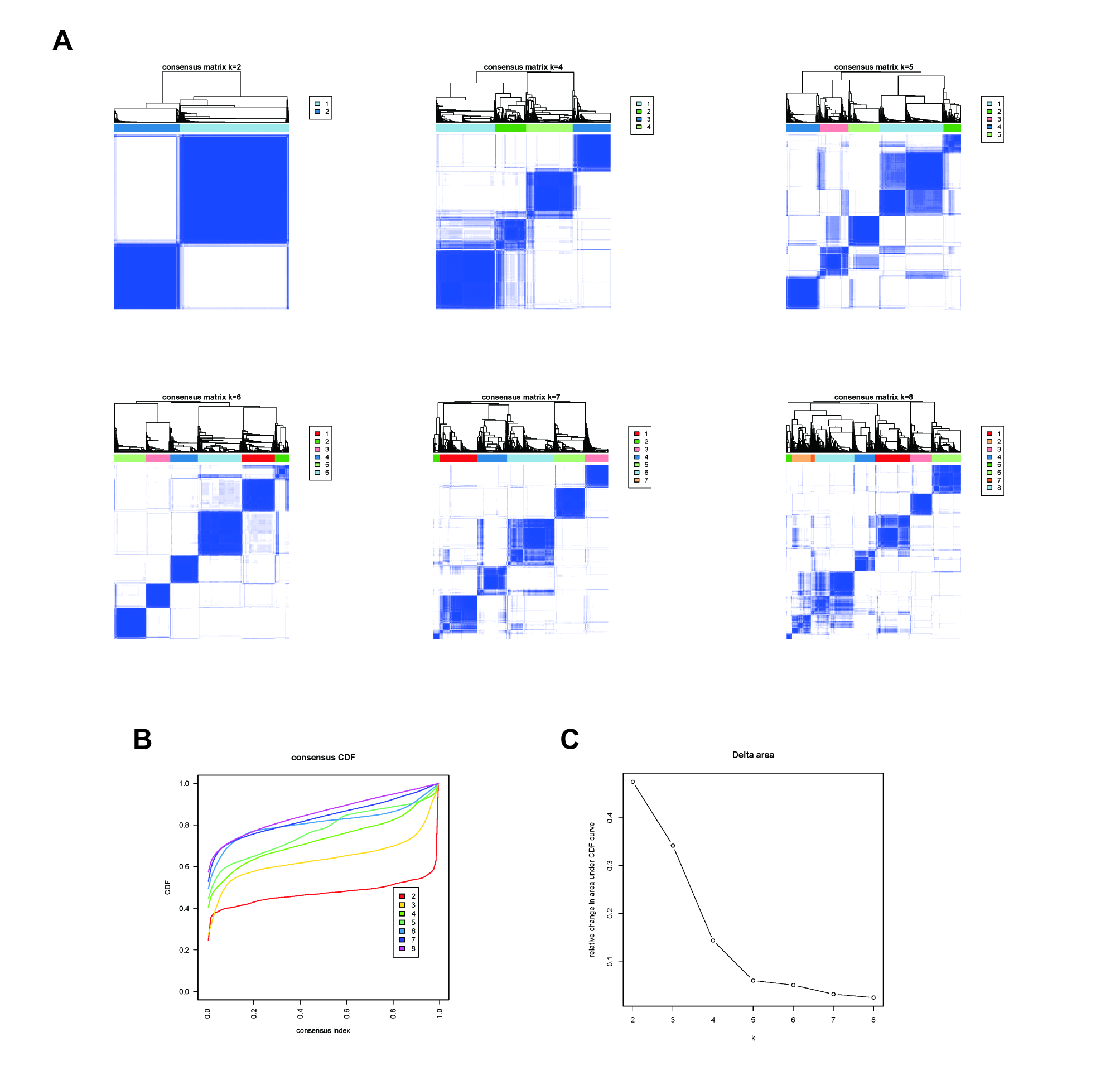

Supplement: Supplementary Figure 1 — Consensus clustering of the glycolysis-related genes. (A) The heatmap of consensus clustering with k from 2 to 8. (B) The CDF threshold curve. (C) The delta area. [file Image_1.tif]

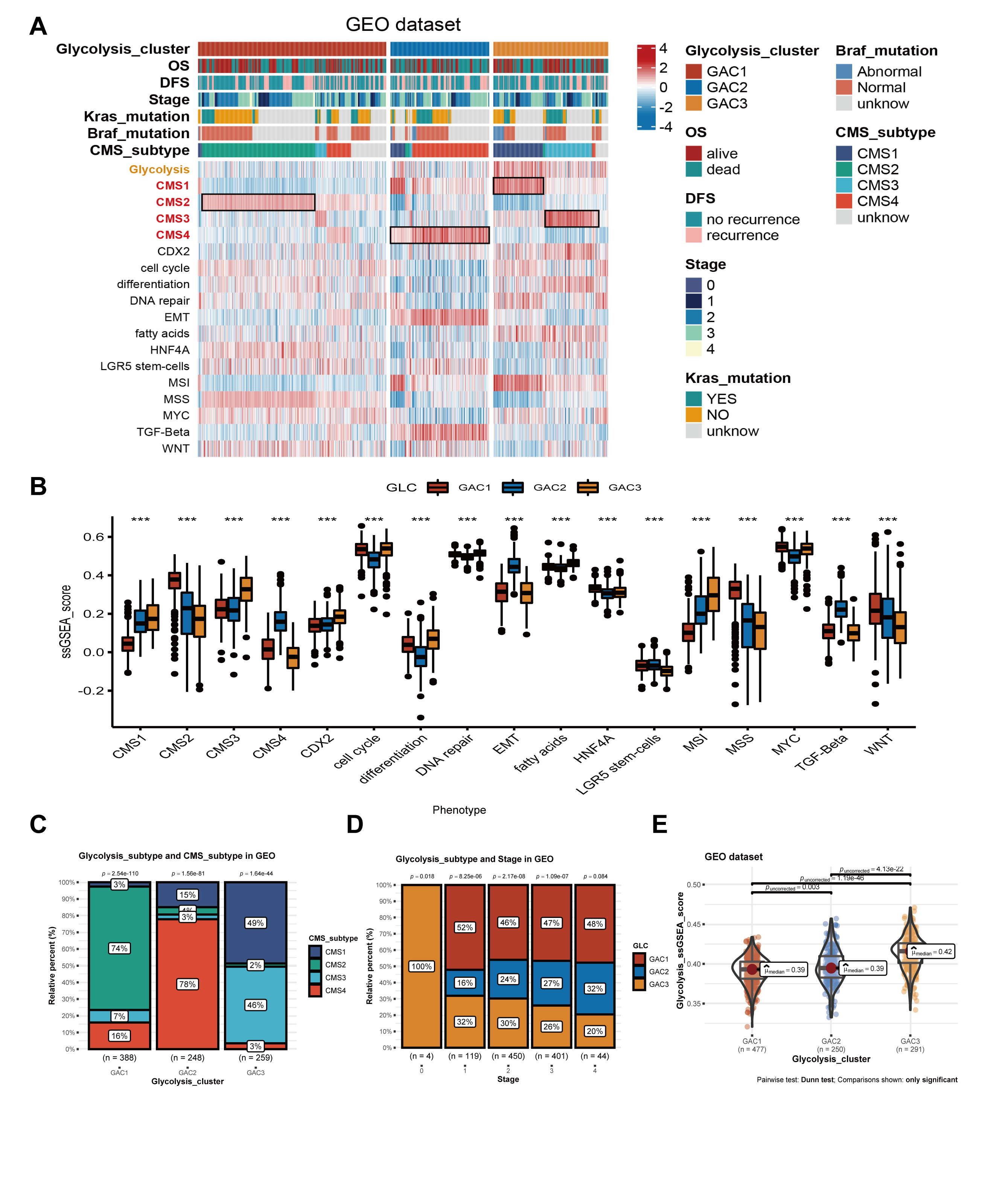

Supplement: Supplementary Figure 2 — The validation of clinical and biological pathway characteristics in GEO datasets. (A) The heatmap comprehensively assessed each GAC’s biological and clinical parameters in GEO datasets. (B) The boxplots visualizing each GAC’s pathway enrichment scores (* p<0.05, ** p<0.01, *** p<0.001). (C) The relationship between GACs and CMS type in GEO datasets. (D) The relationship between GACs and stage in GEO datasets. (E) The distribution of glycolysis score in each GAC. [file Image_2.tif]

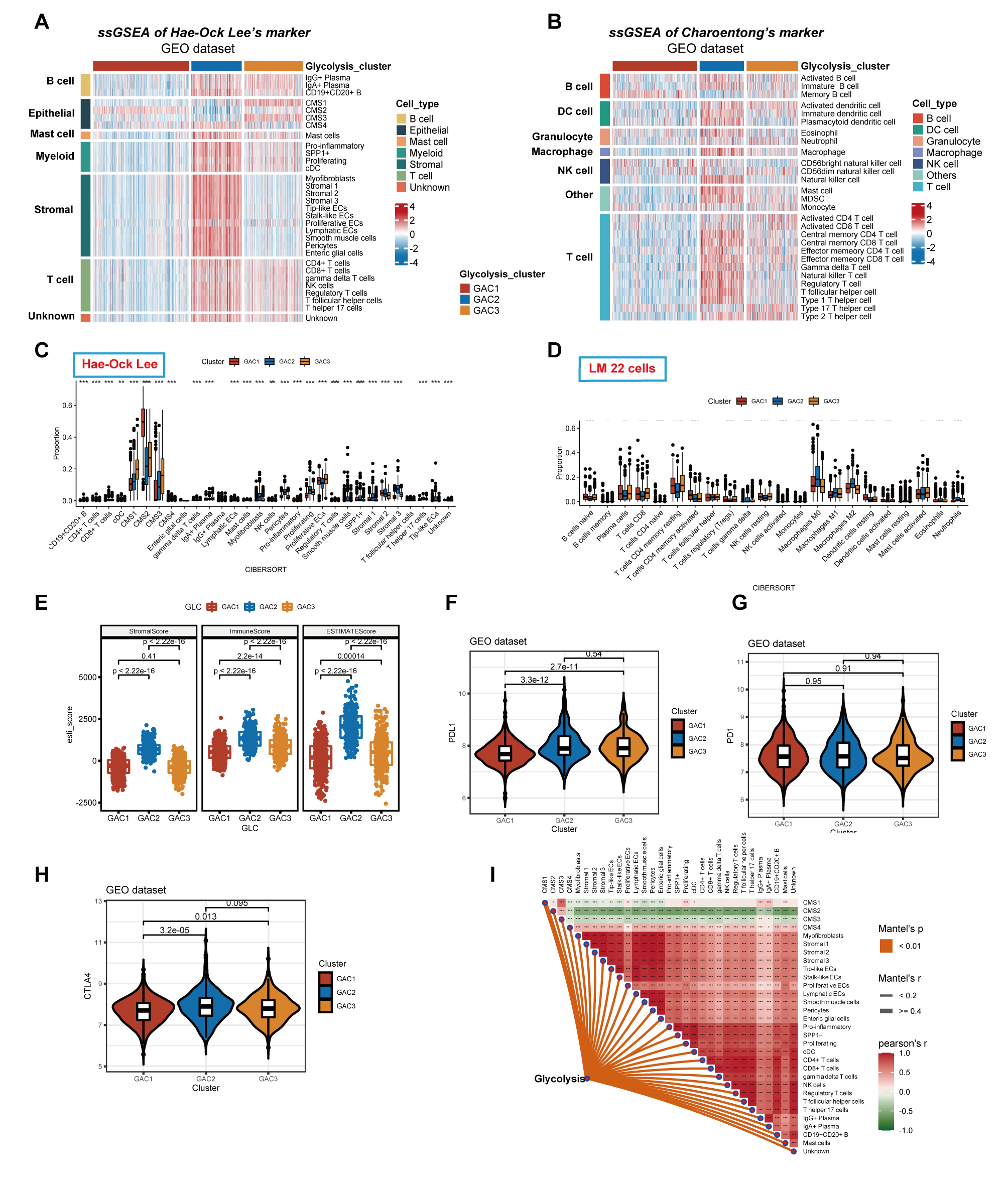

Supplement: Supplementary Figure 3 — The validation of TME features of each GAC in GEO datasets. (A, B) ssGSEA analyses based on markers of Hae-ock Lee and Charoentong revealing the expression of TME cells of the GACs in GEO datasets. (C, D) Cibersort analysis depicting the percentage of each cell in GACs based on markers of Hae-ock Lee and LM 22 cells in GEO datasets (* p<0.05, ** p<0.01, *** p<0.001). (E) Estimate algorithm calculating stromal, immune and overall score of the GACs in GEO datasets. (F–H) The expression of PD1, PDL1, and CTLA4 in each GAC in GEO datasets. (I) The relationship between TME cells and glycolysis score. [file Image_3.tif]

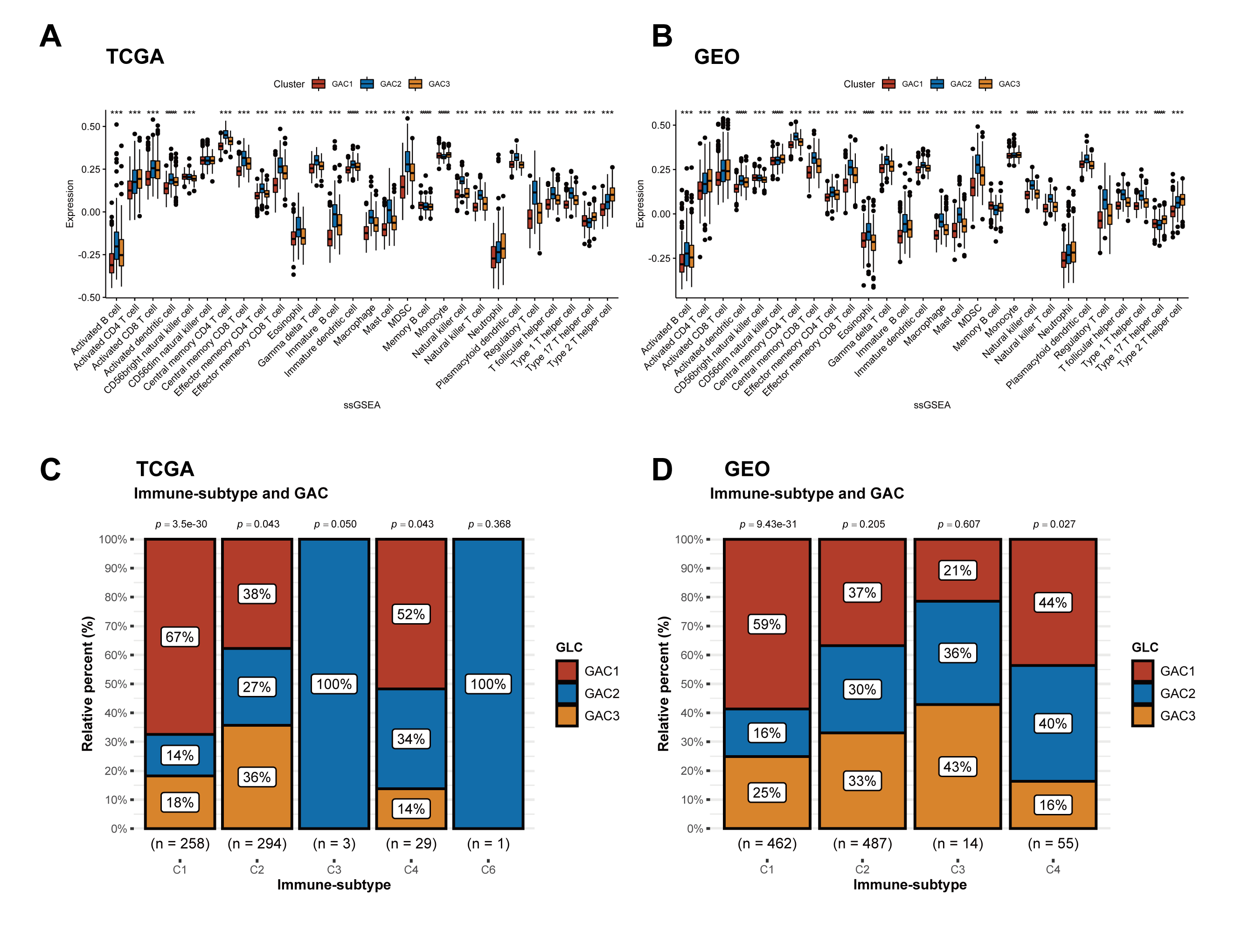

Supplement: Supplementary Figure 4 — Further exploration of immune infiltration of GACs in both TCGA and GEO datasets. (A, B) The boxplots quantifying the ssGSEA score of immune cells in each GAC for the validation. (C, D) The proportion of GACs in each immune-subtype. [file Image_4.tif]

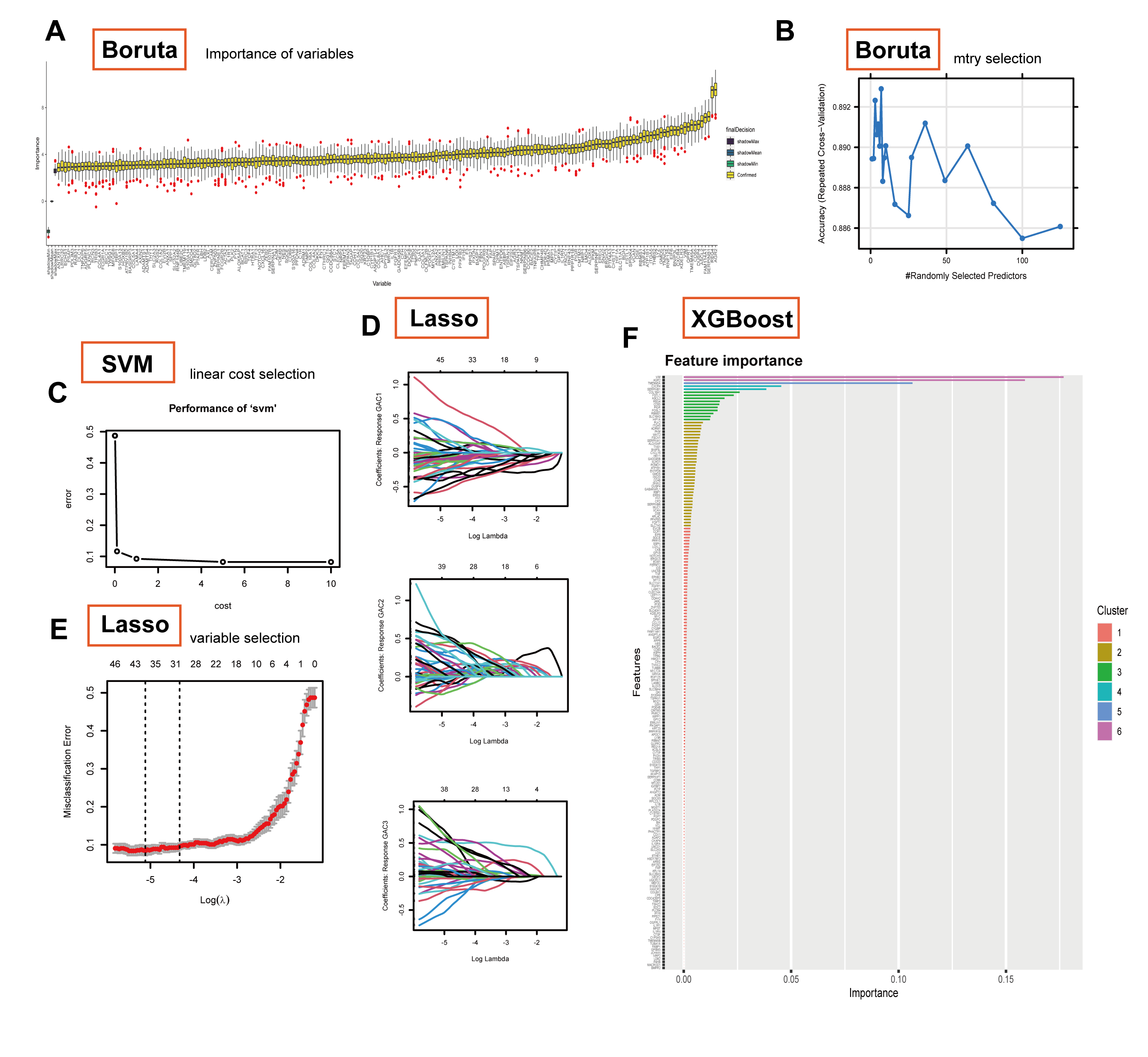

Supplement: Supplementary Figure 5 — The visualization of four machine learning methods contributing to the selection of important genes. (A, B) The importance of the selected features using random forest (Boruta) methods. (C) The linear cost selection of SVM methods. (D, E) The Lasso method filtering variables. (F) The importance of the selected variables using XGBoost method. [file Image_5.tif]
